# Supplementary material for: High-resolution myelin-water fraction and quantitative relaxation mapping using 3D ViSTa-MR fingerprinting
Source: ArXiv. 2023 Dec 21:arXiv:2312.13523v1. Preprint. [Version 1] (PMC10775347)
Supplement: 1 [file NIHPP2312.13523V1-supplement-1.pdf]

Supporting Information

Table S1. MWF comparison between the proposed ViSTa-MRF method and the literature values in splenium, forceps Major/minor, and genu corpus callosum regions.

	Literature values (Ref. (25))	ViSTa-MRF MWF
Splenium	5.4%±0.3%	5.8%±1.2%
Genu	5.8%±0.4%	5.7%±0.8%
Forceps Major	5.2%±0.4%	5.2%±1.0%
Forceps Minor	5.0%±0.4%	5.3%±1.0%

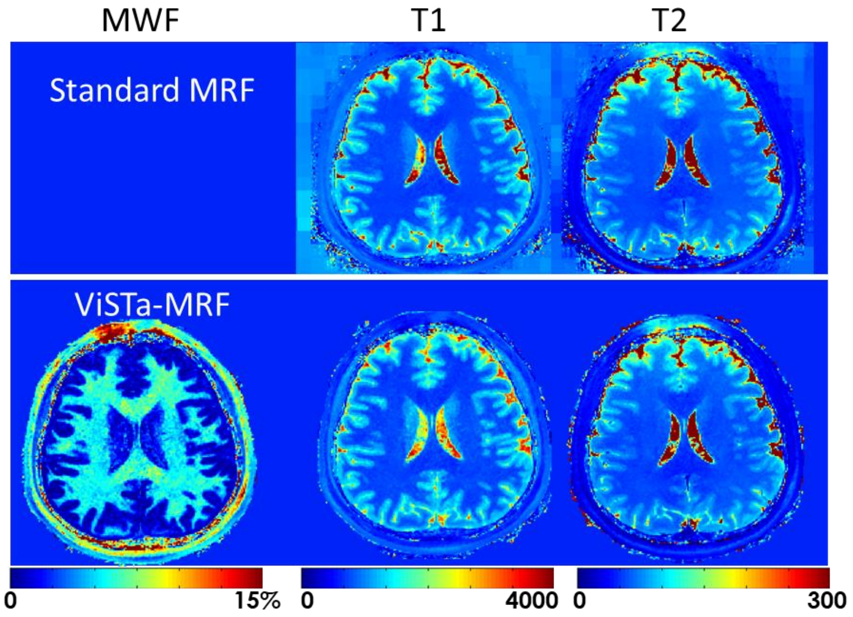

Figure S1. T_1 and T_2 comparison between 1mm-iso ViSTa-MRF and standard MRF.

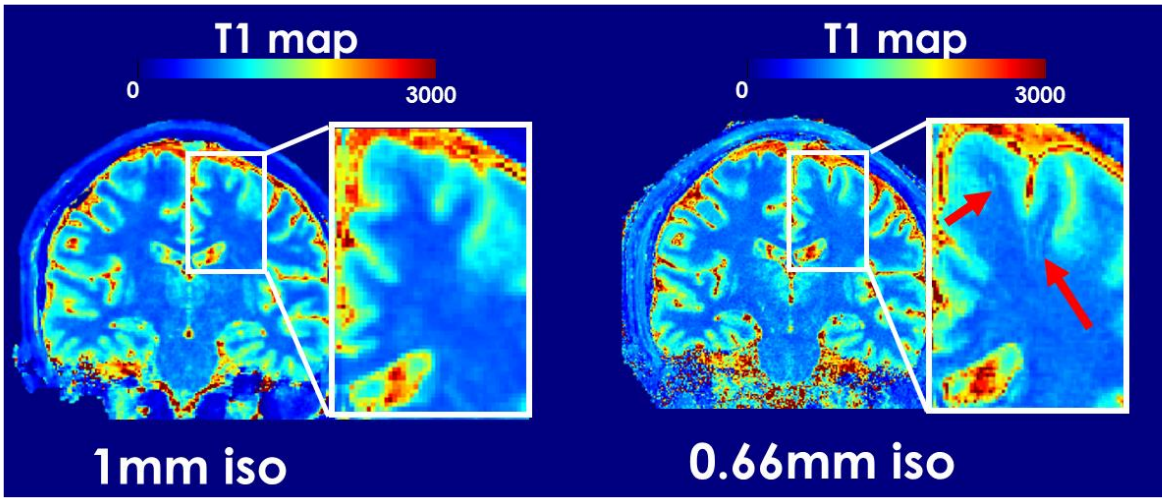

Figure S2. T_1 comparison between 1mm and 0.66-mm data. The higher resolution in the 0.66-mm dataset can aid in better visualization of subtle brain structures such as small sulci and the periventricular space as indicated by the red arrows.

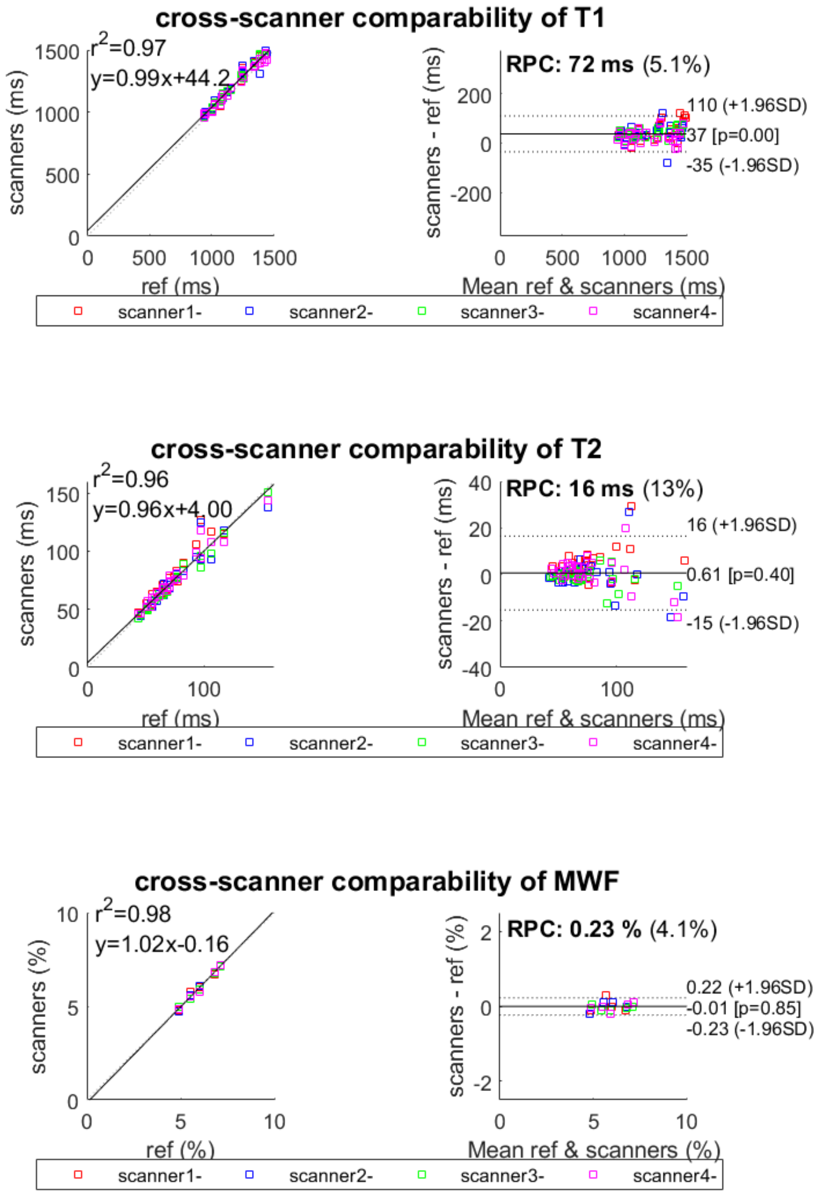

Figure S3. Cross-scanner comparability of ViSTa-MRF results.
